# Supplementary material for: Dredging activity and associated sound have negligible effects on adult Atlantic sturgeon migration to spawning habitat in a large coastal river
Source: PLoS One. 2020 Mar 6;15(3):e0230029. doi: 10.1371/journal.pone.0230029 (PMC7059921; doi:10.1371/journal.pone.0230029)

PLOS ONE

Dredging activity and associated sound have negligible effects on adult Atlantic sturgeon migration to spawning habitat in a large coastal river

--Manuscript Draft--

**Manuscript Number:**	PONE-D-19-29177
**Article Type:**	Research Article
**Full Title:**	Dredging activity and associated sound have negligible effects on adult Atlantic sturgeon migration to spawning habitat in a large coastal river
**Short Title:**	Dredging has negligible effects on sturgeon migration
**Corresponding Author:**	Matthew T. Balazik<br>Engineer Research and Development Center<br>Vicksburg, Mississippi UNITED STATES
**Keywords:**	sturgeon; endangered species; dredging
**Abstract:**	Dredging is considered a major threat/impedance to anadromous fish migrating to spawning habitat. Due to the perceived threat caused by dredging, environmental windows that restrict dredge operations are enforced within many rivers along the east coast. However, it is generally unknown how anadromous fish react to encountering an active dredge during spawning migrations. Atlantic sturgeon (ATS) are an endangered, anadromous species along the Atlantic slope of North America. To determine if and how an active dredge may affect ATS spawning migration, a Vemco Positioning System array was deployed around an active hydraulic-cutterhead dredge that adult ATS must traverse to reach spawning habitat in the James River, VA. Telemetry data showed that all ATS that entered the study area survived. ATS that migrated upstream during dredge operations (N=103) traversed the dredge area and continued upstream to spawning habitat. Many ATS made multiple trips through the study area during dredge operations. There was no noticeable difference in swim behavior regardless of whether the dredge was absent or working within the study area. We suggest that dredging in the lower James River does not create a barrier for adult ATS migrating to spawning habitat or cause adults to significantly modify swim behavior. This is the first study to utilize fine-scale telemetry data to describe how an organism moves in relation to an active dredge. This methodology could be used to describe dredge-sturgeon interactions on different life stages and in other locations and could be expanded to other aquatic organisms of concern.
**Order of Authors:**	Matthew T. Balazik
	Michael Barber
	Safra Altman
	Kevin Reine
	Alan Katzenmeyer
	Aaron Bunch
	Greg Garman
**Additional Information:**	
**Question**	**Response**
**Financial Disclosure**<br><br>Enter a financial disclosure statement that describes the sources of funding for the work included in this submission. Review the submission guidelines for detailed requirements. View published research articles from *PLOS ONE* for specific	MTB, GCG, #NA13NMF4720037 National Oceanic and Atmospheric Administration, Virginia Department of Game and Inland Fisheries. Yes, preparation of the manuscript

Powered by Editorial Manager® and ProduXion Manager® from Aries Systems Corporation

examples.<br><br>This statement is required for submission and **will appear in the published article** if the submission is accepted. Please make sure it is accurate.<br><br>**Unfunded studies**<br>Enter: *The author(s) received no specific funding for this work.*<br><br>**Funded studies**<br>Enter a statement with the following details:<br>• Initials of the authors who received each award<br>• Grant numbers awarded to each author<br>• The full name of each funder<br>• URL of each funder website<br>• Did the sponsors or funders play any role in the study design, data collection and analysis, decision to publish, or preparation of the manuscript?<br>• **NO** - Include this sentence at the end of your statement: *The funders had no role in study design, data collection and analysis, decision to publish, or preparation of the manuscript.*<br>• **YES** - Specify the role(s) played.<br><br>* typeset	
**Competing Interests**<br><br>Use the instructions below to enter a competing interest statement for this submission. On behalf of all authors, disclose any competing interests that could be perceived to bias this work—acknowledging all financial support and any other relevant financial or non-financial competing interests.<br><br>This statement **will appear in the published article** if the submission is accepted. Please make sure it is accurate. View published research articles from *PLOS ONE* for specific examples.	No

**NO authors have competing interests**<br><br>Enter: *The authors have declared that no competing interests exist.*<br><br>**Authors with competing interests**<br><br>Enter competing interest details beginning with this statement:<br><br>*I have read the journal's policy and the authors of this manuscript have the following competing interests: [insert competing interests here]*<br><br>* typeset	
**Ethics Statement**<br><br>Enter an ethics statement for this submission. This statement is required if the study involved:<br><br>• Human participants<br>• Human specimens or tissue<br>• Vertebrate animals or cephalopods<br>• Vertebrate embryos or tissues<br>• Field research<br><br>Write "N/A" if the submission does not require an ethics statement.<br><br>General guidance is provided below. Consult the submission guidelines for detailed instructions. **Make sure that all information entered here is included in the Methods section of the manuscript.**	This study was carried out in guidelines set by Virginia Commonwealth University's Institutional Animal care and Use Committee (#AD20127) and National Marine fisheries Service endangered species permit (#16547).

Powered by Editorial Manager® and ProduXion Manager® from Aries Systems Corporation

**Format for specific study types**<br><br>**Human Subject Research (involving human participants and/or tissue)**<br>• Give the name of the institutional review board or ethics committee that approved the study<br>• Include the approval number and/or a statement indicating approval of this research<br>• Indicate the form of consent obtained (written/oral) or the reason that consent was not obtained (e.g. the data were analyzed anonymously)<br><br>**Animal Research (involving vertebrate animals, embryos or tissues)**<br>• Provide the name of the Institutional Animal Care and Use Committee (IACUC) or other relevant ethics board that reviewed the study protocol, and indicate whether they approved this research or granted a formal waiver of ethical approval<br>• Include an approval number if one was obtained<br>• If the study involved *non-human primates*, add *additional details* about animal welfare and steps taken to ameliorate suffering<br>• If anesthesia, euthanasia, or any kind of animal sacrifice is part of the study, include briefly which substances and/or methods were applied<br><br>**Field Research**<br><br>Include the following details if this study involves the collection of plant, animal, or other materials from a natural setting:<br>• Field permit number<br>• Name of the institution or relevant body that granted permission	
**Data Availability**<br><br>Authors are required to make all data underlying the findings described fully available, without restriction, and from the time of publication. PLOS allows rare exceptions to address legal and ethical concerns. See the PLOS Data Policy and FAQ for detailed information.	Yes - all data are fully available without restriction

A Data Availability Statement describing where the data can be found is required at submission. Your answers to this question constitute the Data Availability Statement and **will be published in the article**, if accepted.<br><br>**Important:** Stating 'data available on request from the author' is not sufficient. If your data are only available upon request, select 'No' for the first question and explain your exceptional situation in the text box.<br><br>Do the authors confirm that all data underlying the findings described in their manuscript are fully available without restriction?	
**Describe where the data may be found in full sentences. If you are copying our sample text, replace any instances of XXX with the appropriate details.**<br><br>• If the data are **held or will be held in a public repository**, include URLs, accession numbers or DOIs. If this information will only be available after acceptance, indicate this by ticking the box below. For example: *All XXX files are available from the XXX database (accession number(s) XXX, XXX.*).<br>• If the data are all contained **within the manuscript and/or Supporting Information files**, enter the following: *All relevant data are within the manuscript and its Supporting Information files.*<br>• If neither of these applies but you are able to provide **details of access elsewhere**, with or without limitations, please do so. For example:<br><br>*Data cannot be shared publicly because of [XXX]. Data are available from the XXX Institutional Data Access / Ethics Committee (contact via XXX) for researchers who meet the criteria for access to confidential data.*<br><br>*The data underlying the results presented in the study are available from (include the name of the third party*	Data can be attained by contacting the corresponding author

and contact information or URL).<br>• This text is appropriate if the data are owned by a third party and authors do not have permission to share the data.<br><br>* typeset	
Additional data availability information:	

Powered by Editorial Manager® and ProduXion Manager® from Aries Systems Corporation

Manuscript

Click here to download Manuscript Balazik et al PloS1_JA_1_Final.docx

Dredging activity and associated sound have negligible on adult Atlantic sturgeon Migration to spawning habitat in a large coastal river

Matthew Balazik[1,2*], Michael Barber[2], Safra Altman[1], Kevin Reine[1], Alan Katzenmeyer[1], Aaron J. Bunch[3], Greg Garman[2]

[1] Engineer Research and Development Center, United States Army Corps of Engineers, Vicksburg, MS

[2] Rice Rivers Center, Virginia Commonwealth University, Charles City, VA

[3]Virginia Department of Game and Inland Fisheries, Charles City, VA

* Corresponding author

E-mail: Matthew.T.Balazik@usace.army.mil

Abstract

Dredging is considered a major threat/impedance to anadromous fish migrating to spawning habitat. Due to the perceived threat caused by dredging, environmental windows that restrict dredge operations are enforced within many rivers along the east coast. However, it is generally unknown how anadromous fish react to encountering an active dredge during spawning migrations. Atlantic sturgeon (ATS) are an endangered, anadromous species along the Atlantic slope of North America. To determine if and how an active dredge may affect ATS spawning migration, a Vemco Positioning System array was deployed around an active hydraulic-cutterhead dredge that adult ATS must traverse to reach spawning habitat in the James River, VA. Telemetry data showed that all ATS that entered the study area survived. ATS that migrated upstream during dredge operations (N=103) traversed the dredge area and continued upstream to spawning habitat. Many ATS made multiple trips through the study area during dredge operations. There was no noticeable difference in swim behavior regardless of whether the dredge was absent or working within the study area. We suggest that dredging in the lower James River does not create a barrier for adult ATS migrating to spawning habitat or cause adults to significantly modify swim behavior. This is the first study to utilize fine-scale telemetry data to describe how an organism moves in relation to an active dredge. This methodology could be used to describe dredge-sturgeon interactions on different life stages and in other locations and could be expanded to other aquatic organisms of concern.

Introduction

Through Coastal Zone Management Acts, many states have implemented time-of-year restriction windows, or environmental windows, on dredging projects. Environmental windows are in place to minimize possible negative impacts that dredging projects may have on natural resources. In 2012, the National Marine Fisheries Service declared that distinct population segments of Atlantic sturgeon *Acipenser oxyrinchus oxyrinchus* (ATS) were either threatened or endangered throughout their entire range along the Atlantic coast [1-2]. ATS are both anadromous and philopatric species meaning they return to upstream freshwater reaches in their natal streams to spawn [3]. Currently, areas along the U.S. east coast have established riverine environmental windows for the spring anadromous fish spawning run. When anadromous fish windows were implemented it was thought that ATS spawning was limited to the spring season. However, studies have shown that in some river systems, ATS spawning occurs during both the spring and fall seasons [4-9]. There are no riverine environmental windows currently in place to protect fall spawning ATS migrating to spawning habitat. Although the supporting data are sparse, dredging is listed as a serious threat to ATS recovery [3] and resource managers are concerned about potential impacts an active dredging operation may have on fall run adult ATS migrating to spawning habitat.

Due to shoaling (i.e., buildup of bottom sediments in river channels) in the federal navigation channel, dredging occurs annually between river kilometer (rkm) 40 and 60 of the James River, Virginia. To create safe passage for shipping, dredging typically begins soon after the spring anadromous fish environmental window ends in July and dredging continues through November. Fall spawning ATS usually stage in the lower part of the James River near rkm 30 until water temperatures

decrease below 28°C during August-September and then move upstream above rkm 130 to spawn [7,10]. This creates a situation where adult ATS must traverse annual dredging operations to reach spawning habitat. The goal of this study to determine how migrating adult ATS move in relation to an active hydraulic-cutterhead dredge by utilizing Vemco Positioning System (VPS) technology. Implications of this work will help determine if environmental windows are an effective management policy.

METHODS

Study Area

The study area is a 12 km reach of the James River federal navigation channel (Fig 1). Due to severe shoaling in the federal navigation channel, dredging occurs annually from July through November to maintain legally required channel depths. In 2017 two portions of the 12 km stretch were dredged (Fig 1).

Fig 1. Map of study design. The white polygon is the navigation channel and the colored dots within the channel are where the hydraulic-cutterhead dredge was working during the study period. The black dots are collocated receiver locations of the Vemco Positioning System array. The red dot is a reference tag to assist with determining fine-scale positions for the telemetered Atlantic sturgeon. The gray dots are receiver locations for the supplemental array upstream of the dredge area. The black grid in the southern area of the array is where the dredged material from the lower dredge area was placed.

Vemco Positioning System Array

A VPS utilizes trilateralization technology to estimate down to 1 m spatial resolution of telemetered objects within a study area [11]. This technique requires acoustic receivers to be strategically placed to allow a single ping from a telemetry tag to be detected by at least three receivers. Using difference in arrival time to multiple receivers the position of the tag can be estimated. The tagged organisms in this study were telemetered fall spawning ATS. During previous years, Virginia Commonwealth University has surgically implanted Vemco telemetry tags into various sized ATS in the James River [5,10]. At the time of this project there were about 140 adult James River population ATS at large of which about 100 enter the James River annually to spawn.

A 37 bottom mounted VPS receiver array with collocated synchronization tags was deployed around rkm 55 to monitor movements of telemetered ATS in the lower dredge area (Fig 1). One reference tag was placed in the middle of the array to improve positioning data of the ATS (Fig 1). It is very important to minimize receiver movements when conducting VPS studies [11]. VPS Receivers were placed inside 0.7-1 m long, 8 cm diameter PVC pipes with the receiver hydrophone exposed on one end with the other end of the pipe embedded in a 61X61X12 cm concrete slab weighing about 45kg (Fig 2). Rebar cross members that extend about 50 cm from each direction of the concrete slab were added to strengthen the concrete. The reinforced concrete slab and extended rebar helped prevent the receiver from moving or tipping over in the water current. Rebar bent in a U-shape was placed in the concrete to provide handles to make moving the receiver stands easier and also create a grappling point. A 30 m piece of positively buoyant rope was secured to the receiver stand and the other end of the rope was secured to a 2 kg anchor or cinder block (Fig 2). ~~The rope allowed~~ the gear to be removed using a grappling hook. The VPS array deployment was initiated on July 20, completed on August 6 and was removed October 29, 2017.

Fig 2. Receiver setup to reduce movements of receives in the Vemco Positioning System array.

To provide additional receiver coverage at the upper dredge area, a supplemental receiver gate comprising 4 receivers was placed on July 20 about 2 km upstream of the upper dredge area (Fig 1). Each supplemental receiver was affixed to the bottom with an 11 kg anchor. The supplemental gate could not determine fine-scale positions, but could determine when adult ATS passed the upper dredge area.

Position Analysis

VPS positions used for data analysis were vetted by only using positions estimated to be within the array grid (Fig 3). Positions were vetted further following methods by Roy et al. [11] using horizontal positioning error scores. The Optimized Hot Spot Analysis tool (ESRI ArcMap 10.5.1 Pro) was used to determine areas that ATS more frequently used while moving through the study area. The Optimized Hot Spot Analysis tool removes the subjective aspect of determining point clusters by assigning confidence values to each cell as either a significant hot spot, a non-significant spot, or a significant cold spot. This technique utilizes inferential statistics that applies *z* and *p* scores to hexagon polygons using Getis-Ord Gi* Cluster Analysis [12]. The hotspot analysis was confined to the rectangular bounding grid used for vetting the VPS positions (Fig 3). Position data was divided into three timeframes: 1) dredge operational in lower area, 2) dredge operational in upper area, and 3) no dredge operations occurring.

Figure 3. Points showing all adult Atlantic sturgeon positions generated by the Vemco Position System array. The black box is the extent of the Getis Gi* Hotspot analysis. In total 99 adults were

represented and 5839 positions were recorded of which 5,011 were used after the points were vetted for accuracy.

Other VPS studies have been used to describe sturgeon swim behavior [13,14]. Not all fish paths were used for swim behavior analysis. Only fish tracks extending at least 1 km with no consecutive points greater than 15 minutes apart were used for the swim behavior comparisons. Each path was classified according to whether the direction of movement was upstream or downstream, as well as whether the dredge was at the downstream site, the upstream site or absent. Swim speeds were determined by measuring the distance between all points of an individual track and dividing by elapsed time. Permutation tests with 25,000 iterations using the 'independence_test' function in R Statistical Software, version 3.5.2, package "coin 1.3-0" were run comparing ATS swim speed versus movement direction through the study area and whether the dredge was working in the lower area, upper area, or if the dredge was absent [15]. Another aspect of swim behavior analyzed was how much an ATS deviated from a straight swam path, *i.e.* meandered, while moving through the study area. The difference in the actual distance swam and the straight line distance between the first and last point of the track was calculated in meters. Using the same methods as the speed permutation test, separate permutation tests were run on how much ATS meandered while traversing through the study area.

Water Quality

Water temperature is a critical factor influencing the timing of ATS migration to spawning habitat [16]. ~~The National Oceanic and Atmospheric Administration operates a water quality buoy less than 2 km downstream of the study area that takes a suite of hourly measurements [17].~~ Average

hourly water temperature measurements were estimated from July 20 to November 3, 2017. In previous years, male fall spawning ATS tended to start migrating upstream when the daily mean water temperatures fell below 29°C (Matthew Balazik, Virginia Commonwealth University, Unpublished Data).

Dredge

The hydraulic-cutterhead dredge, *Lexington*, conducted maintenance dredging during the study period. The *Lexington* is 61 m long, 125 m wide, 3 m draft barge with a 2000 HP pump connected to a 51 cm diameter intake pipe located just shipward of the cutterhead. The cutterhead tip had a 1.4 m diameter (Fig 4). Dredge operations started at 1500 on July 27 and where continuous until the project ended at 1700 on September 16, 2017. Dredging occurred in the lower area from July 27 to August 23 and September 12 to 14. Dredging occurred in the upper area from August 23 to September 12 and September 15 and 16 (Figure 5). The dredge removed 278,056 m^3 of sediment from the lower dredge area and 227,311 m^3 from the upper area (Fig 5). The dredge's location was determined at 30 s intervals using the United States Army Core of Engineers Automatic Identification System Analysis Package (AISAP) tool. All dredged material was placed via a partially floating and sinking pipeline to a pre-determined site located south of the navigation channel (Fig 1).

Figure 4. The top panel shows the hydraulic-cutterhead dredge *Lexington*. The bottom panel is a picture of the actual cutter head used during the project.

Figure 5. The primary Y-axis shows how much material was removed from the lower (solid gray line) and upper (dashed gray line) dredge area. The secondary Y-axis shows temperature (red line) and number of adult Atlantic sturgeon moving passed the dredge or through the area (black line) each day.

Serendipitously dredging started when water temperatures dropped to levels that trigger upstream spawning migrations.

RESULTS AND DISCUSSION

During the study period 106 adult ATS (7 female and 99 male) were detected in the study area, 103 of which swam past the active dredge. No ATS moved through the VPS array without being detected. Accurate positions could not be determined for 7 males, but the dates of movement are known. A total of 5,839 fine-scale positions were determined by the VPS of which 5,011 from 7 females and 92 males were used for analysis (Fig 3). All ATS were detected by the supplemental array. The 7 males that had no fine-scale positions estimated moved upstream in late August during the peak of upstream migration (Fig 5). During dredging operations in the lower area, 48 different adult ATS made a total of 88 passes by the active dredge (Fig 5). Even though the VPS did not provide coverage for the upper dredge area it can be determined whether a fish moved past the dredge by combining detections from the VPS array and the supplemental upstream receiver gate (Fig 1). Seventy-four adults moved past the dredge a total of 129 times while the dredge was working in the upper area (Fig 5). After dredging finished on September 16, 93 adults traversed the study area 125 times (Fig 5).

Three male ATS moved through and staged upstream of the study area prior to dredge operations. As in previous years, the majority of adults started moving upstream when daily mean water temperatures at the water quality buoy fell below 29°C (Fig 5). Mean daily water temperatures fell below 29°C in the study area on July 30. ATS staging downstream started migrating upstream to spawning habitat on July 29 when the dredge was working in the lower area. Adult activity gradually increased in the study area but then decreased as water temperatures began increasing around August

16 (Fig 5). As water temperatures dropped relatively quickly starting August 28, upstream migration activity peaked while the dredge was working in the upper area (Fig 5). Based on real-time receivers around hypothesized spawning habitat, the peak of the 2017 spawning occurred from September 5 to 14. When dredging operations were finishing in the upper area there was a spike in migrating adults on September 15 as the post-spawn ATS began to return to the ocean. Most telemetered male ATS stayed around spawning habitat for weeks after the peak spawning season, but started migrating downstream in early October (Fig 5).

The dredge did not limit ATS movement or cause mortalities. No ATS that reached the study area while the dredge was working failed to move upstream ~~past~~ the dredge. Telemetry data showed that ATS swam past dredge operations 217 times. Over half (n = 59, 56%) of the spawning ATS made one pass by the dredge. Forty-four adults (41%) passed the active dredge multiple times with one ATS passing the dredge 13 times (Fig 6). If the active dredge and associated noise were a deterrent it is unlikely adult ATS would make unnecessary, repeat trips past the dredge. Three males ~~never moved past the active dredge because they~~ moved upstream in June before dredge operations and downstream in October, completely missing the dredge activity altogether (Fig 6).

Figure 6. The number of times adult Atlantic sturgeon moved past the hydraulic-cutterhead dredge. The Y-axis is the total number of fish that passed the dredge the number of times listed on the X-axis.

Hot Spot Analysis showed adult positions were more densely packed in the immediate channel area during upstream and downstream migration (Fig 7). When the dredge was working in the lower and upper areas, most of the adults were making upstream migrations to spawning habitat. Adult positions were concentrated in the channel during upstream migration regardless of the presence or

absence of an operating dredge in the immediate area and avoided areas away from the channel (Fig 7). During the main downstream migration after dredging was complete, adult ATS still had high densities in the channel but were more scattered throughout the study area compared to when ATS were mostly moving upstream to spawning habitat (Fig 7).

Figure 7. Results of the Getis Gi* Hotspot Analysis for when the dredge was working in the lower area (Panel A), working in the upper area (Panel B) and when no dredging was occurring (Panel C). The black dots in the middle of the hot spot in Panel A shows where the dredge was working during the lower area. The white polygon marks the federal navigation channel. All three plots show Atlantic sturgeon's preference for the navigation channel either with or without a hydraulic-cutterhead dredge conducting dredge operations.

It is possible that dredge activity attracts migrating adult ATS to the channel, but rather the adult fish naturally utilize the channel during upstream migration due to depth, current flow or some other factors. Catch and telemetry data show migrating adults have a high affinity for deep channels [18] in the James River and results from this study suggest the preference continues even if dredging is occurring. Over the past 5 years, telemetered adult ATS have traversed active dredge operations hundreds of times in the James River and data indicate that no mortalities of telemetered ATS resulted. The VPS telemetry data from this study indicate that no telemetered adults were killed during dredging operations and none stopped upstream progress due to the dredge. Examples of individual fish tracks in relation to dredge are shown in Figure 8.

Figure 8. Individual tracks (colored lines) of adult Atlantic sturgeon swimming in an upstream to spawning habitat during dredge operations. The colored dots represent the dredge location for when the sturgeon of the same color line moved through the area.

ATS traversed the VPS study area 298 times, 170 of which satisfied our criteria for individual movement analysis. ~~All permutation tests resulted in no significant differences in~~ ATS swim speed or meandering when factoring for dredge location and ATS swim direction (Table 1, Fig 9). These data suggest that there was no difference in swim speed or meandering in the VPS area whether the dredge was operational within the lower or upper areas or not operational.

Figure 9. Left boxplots show swim speed (meters per second) and right boxplots show meandering (difference in actual swim path from a linear swim path) when the dredge was not in the area (Absent), when the dredge was working in the lower area (Lower) and when the dredge was working in the upper area (Upper). The top boxplots are when Atlantic sturgeon where swimming upstream through the study area while the bottom boxplots are when Atlantic sturgeon were swimming downstream through the study area. Permutation tests showed no significant difference between swim speed and dredge location for upstream movements ($p=0.429$) and dredge location for downstream movements ($p=0.756$). Permutation tests also showed no significant difference between meandering and dredge location for upstream movements ($p=0.44$) and for dredge location and downstream movements ($p=0.96$).

Table 1. Results of permutation tests comparing swim speed and meandering behavior factoring in swim direction and dredge location.

	Number of tracks	Average swim speed (m/s)	Average meandering (m)

Fish movement direction	Absent	Lower	Upper	Absent	Lower	Upper	p-value	Absent	Lower	Upper	p-value
Upstream	8	34	36	0.71	0.83	0.78	0.43	458	454	297	0.44
Downstream	57	15	20	0.57	0.59	0.67	0.76	472	511	447	0.96

Conclusions

Hydraulic-cutterhead dredging has occurred in the James River during the summer-fall every year since 1998 except for 2000, 2006 and 2013. While dredging is currently considered to be a major threat to recovery, the James River fall ATS population is estimated to be in the thousands and robust compared to most other ATS populations [5,7,18-19]. Based on length-at-age frequencies, most of the current fall adult ATS were born after 1998 [20] indicating that dredge operations has not stopped spawning success in the past. Collections of fall spawn young-of-year and age-1 ATS show successful spawning during the past few years. The results of this study indicate that hydraulic-cutterhead dredging in the study area does not deter adult ATS from migrating to spawning habitat and had no observable effect on swim behavior. This study along with Reine et al. [21] found no evidence of any subadult or adult ATS being killed by a hydraulic-cutterhead dredge in the James River. The combined evidence of this study and past data suggests that the physical presence of a hydraulic-cutterhead dredge and associated underwater sounds in the routinely dredged area during the spawning season does not hinder ATS spawning success.

Data from this study suggest that environmental windows in this area may not be an effective management policy. This study was conducted in a relatively broad, >4 km wide, section of the James River; however, ATS spawning occurs further upstream where the river is typically <200 m wide. While

in this study many adults passed within 100 m of the dredge, there may be a different reaction in a much narrower channel. Mimicking this type study in a narrower channel or closer to spawning habitat would provide useful information for resource managers. More work is also needed to look at potential impacts of various dredge types on juvenile ATS in different environments. It would also be beneficial to conduct fish movement-dredge VPS studies to examine other species of concern, such as striped bass (*Morone saxatilis*) and Alosines, which are protected by the anadromous fish environmental dredge window.

Acknowledgements

We would like to thank Albert Spells (US Fish and Wildlife Service), Eric Hilton, Patrick McGrath and Matthew Fisher (Virginia Institute of Marine Science), Robert Greenlee (Virginia Department of Game and Inland Fisheries), Martin Balazik, Thiwaporn Balazik, George Trice, Kelly Place, and Charles Frederickson (field assistants) and Douglas Clarke (Clarke Environmental) for assisting with this project. This work was partially supported by NOAA Section 6 grant #NA13NMF4720037, the Virginia Department of Games and Inland Fisheries award #NA13NMF4720037. This manuscript represents VCU Rice Rivers Center publication ###. The authors declare that they have no conflict of interest.

REFERENCES

1. USA Federal Register. Endangered and threatened wildlife and plants: final listing for two distinct population segments of Atlantic Sturgeon (*Acipenser oxyrinchus oxyrinchus*) in the southeast. USA Fed Reg. 2012;77: 5914-5982.
2. USA Federal Register. Endangered and threatened wildlife and plants; threatened and endangered status for distinct population segments of Atlantic Sturgeon in the Northeast. USA Fed Reg. 2012;77: 5880-5912.
3. ASSRT. Status review of Atlantic Sturgeon (*Acipenser oxyrinchus oxyrinchus*). Report to the National Marine Fisheries Service, Northeast Regional Office, February 23, 2007. 174 pp.
4. Collins MR, Smith TJ, Post WC, Pashuk O. Habitat utilization and biological characteristics of adult Atlantic sturgeon in two South Carolina Rivers. Trans Am Fish Soc. 2000;129: 982-988.
5. Balazik MT, Garman GC, Van Eenennaam JP, Mohler J, Woods III LC. Empirical evidence of fall spawning by Atlantic sturgeon in the James River, Virginia. Trans Am Fish Soc. 2012; 141: 1465-1471. doi:10.1080/00028487.2012.703157.
6. Smith JA, Flowers HJ, Hightower JE. Fall spawning of Atlantic Sturgeon in the Roanoke River, North Carolina. Trans Am Fish Soc. 2015; 1: 48-54. doi:10.1080/00028487.2014.965344
7. Balazik MT, Musick JA. Dual annual spawning races in Atlantic sturgeon. PloS ONE. 2015;10: e0128234. doi:0128210.0121371/journal.pone.0128234.
8. Balazik MT, Farrae DJ, Darden TL, Garman GC. Genetic differentiation of spring-spawning and fall-spawning male Atlantic sturgeon in the James River, Virginia. PLoS ONE. 2017;12(7): e0179661. doi:10.1371/journal. pone.0179661.

9. Farrae DJ, Post WC, Darden TL. Genetic characterization of Atlantic sturgeon, Acipenser oxyrinchus oxyrinchus, in the Edisto River, South Carolina and identification of genetically discrete fall and spring spawning. Conserv Genet. 2017;18: 1-11. doi:10.1007/s10592-017-0929-7.

10. Balazik MT. Capture and brief invasive procedures using electronarcosis does not appear to affect postrelease habits in male Atlantic sturgeon during the spawning season. N Am J Fish Manag. 2015;35: 398-402. doi:10.1080/02755947.2015.1011358

11. Roy R, Beguin J, Argillier C, Tissot L, Smith F, Smedbol S, De-Oliveira E. Testing the Vemco Positioning System: spatial distribution of the probability of location and the positioning error in a reservoir. Anim Biotele. 2014;2. doi:10.1186/2050-3385-2-1.

12. Getis A, Ord J. The analysis of spatial association of distance statistics. Geogr Anal. 1992;24: 189-206. doi:10.1111/j.1538-4632.1992.tb00261.x.

13. McLean M, Simpfendorfer C, Heupel M, Dadswell M, Stokesbury M. Diversity of behavioural patterns displayed by a summer feeding aggregation of Atlantic sturgeon in the intertidal region of Minas Basin, Bay of Fundy Canada. Mar Ecol Prog Ser. 2014;496: 59-69. doi: 10.3354/meps10555.

14. Hondorp D, Holbrook C, Krueger C. Effects of acoustic tag implantation on lake sturgeon Acipenser fulvescens: lack of evidence for changes in behavior. Anim Biotele. 2015;3: 44-56. doi: 10.1186/s40317-015-0085-0.

15. R Core Team (2018) R: A language and environment for statistical computing. R Foundation for Statistical Computing, Vienna, Austria. https://www.R-project.org/

16. Vine R, Holbrook C, Post W, Peoples B. Identifying environmental cues for Atlantic sturgeon and shortnose sturgeon spawning migrations in the Savannah River. Trans Am Fish Soc. 2019;39: 939-950. doi: 10.1002/tafs.10163

17. https://buoybay.noaa.gov/locations/jamestown

18. Balazik MT, Reine KJ, Spells AJ, Fredrickson CA, Fine ML, Garman GC, McIninch SP. The potential for vessel interactions with adult Atlantic sturgeon in the James River, Virginia. N Am J Fish Manag. 2012;32: 1062-1069. doi: 10.1080/0255947.2015.1011358

19. Hilton E, Kynard B, Balazik M, Horodysky A, Dillman C. Review of the biology, fisheries, and conservation status off the Atlantic sturgeon, (*Acipenser oxyrinchus oxyrinchus* Mitchill, 1815). J Appl Ichthyol. 2016;32: 30-66. doi: 10.1111/jai.13242.

20. Balazik MT, McIninch SP, Garman GC, Latour RJ. Age and growth of Atlantic sturgeon in the James River, Virginia, 1997-2011. Trans Am Fish Soc. 2012;141: 1074-1080. doi: 10.1080/00028487.2012.676590.

21. Reine K, Clarke D, Balazik M, O'Haire S, Dickerson C, Frederickson C, Garman G, et al. Assessing impacts of navigation dredging on Atlantic sturgeon (*Acipenser oxyrinchus*). Technical Report ERDC/EL TR-1412. 2014. doi: 10.13140/RG.2.1.5064.6565.

Figure 1

Click here to download Figure Fig 1.tif

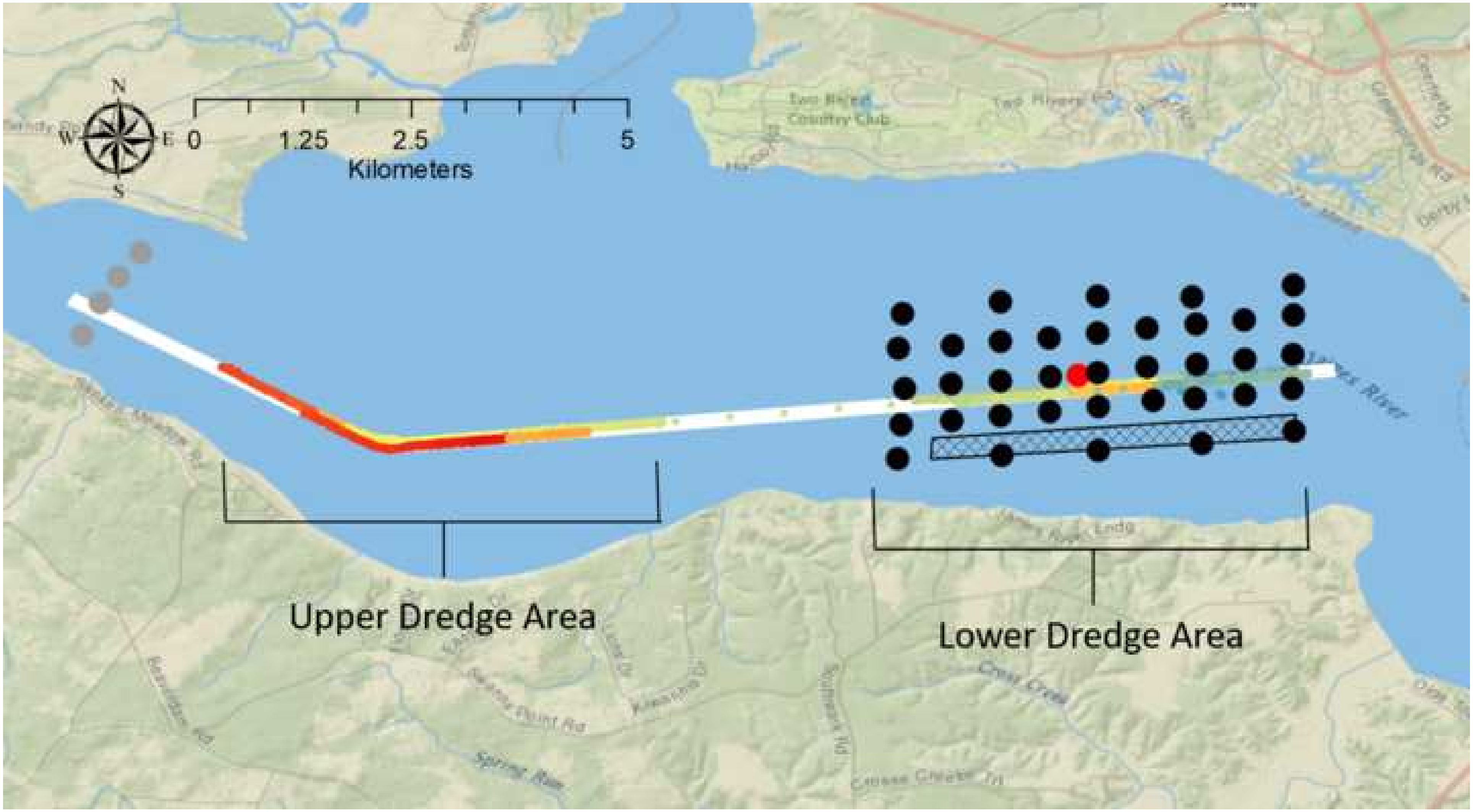

Figure 2

Click here to download Figure Fig 2.tif

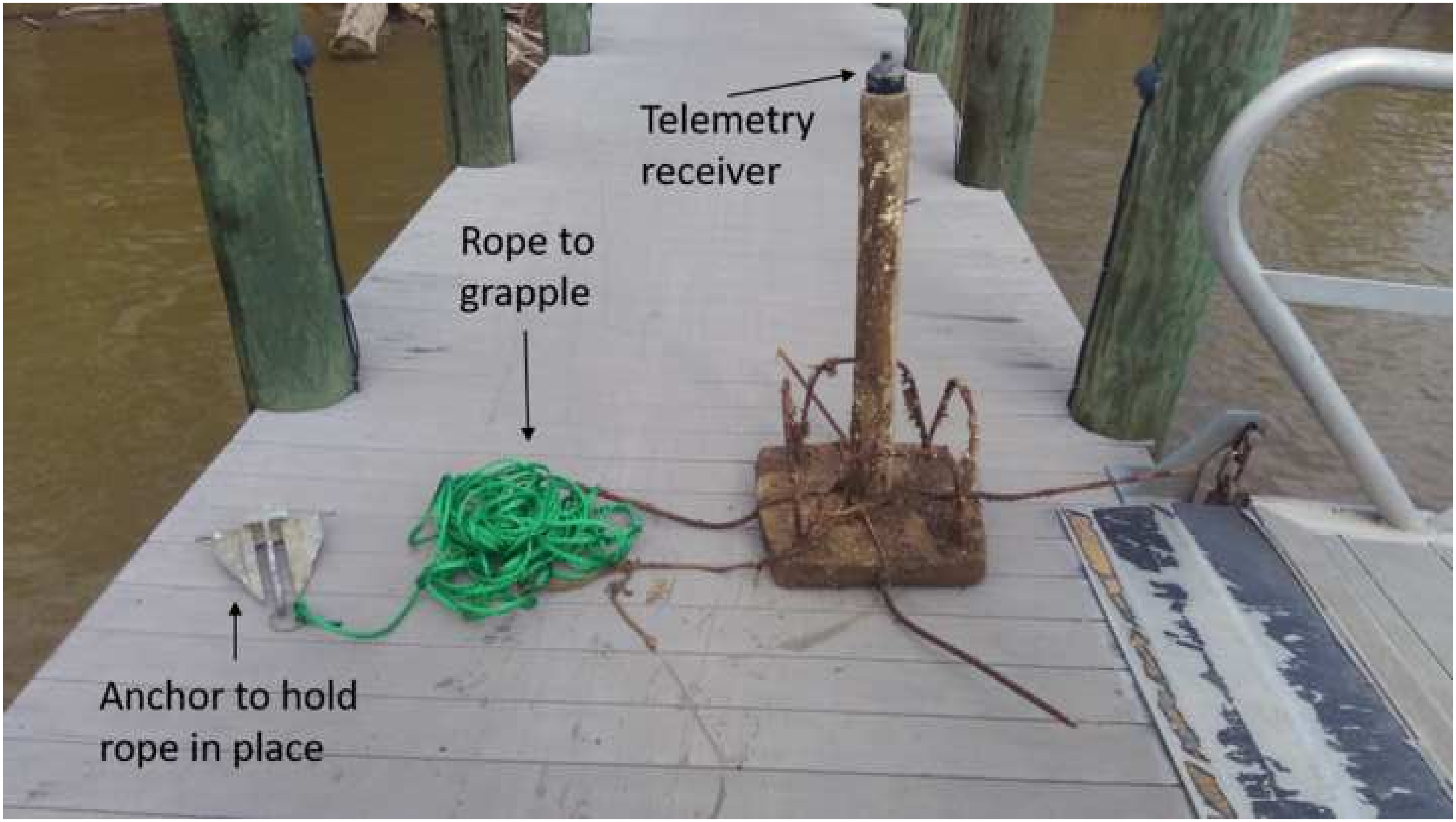

Figure 3

Click here to download Figure Fig 3.tif

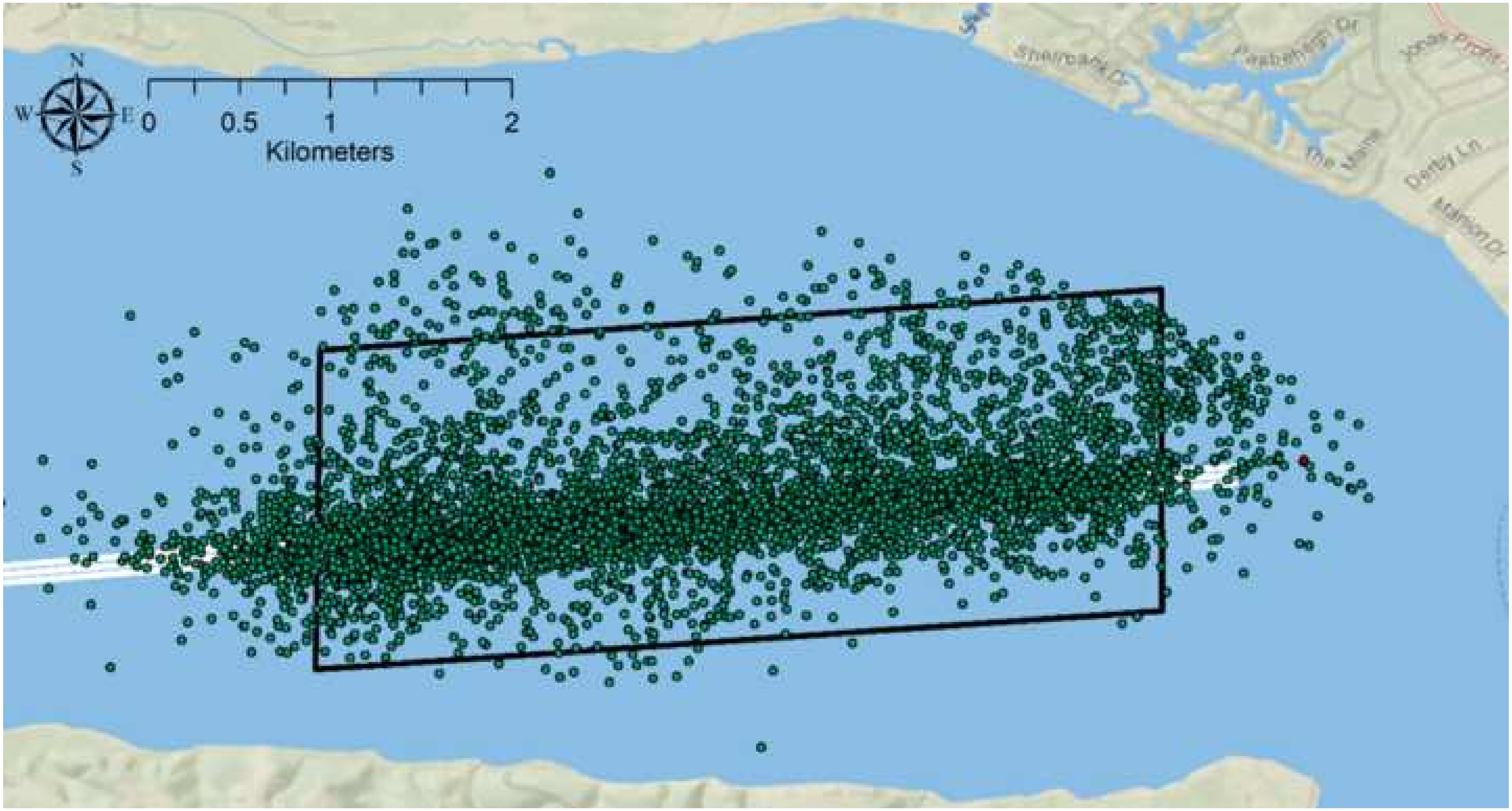

Figure 4

Click here to download Figure Fig 4.tif

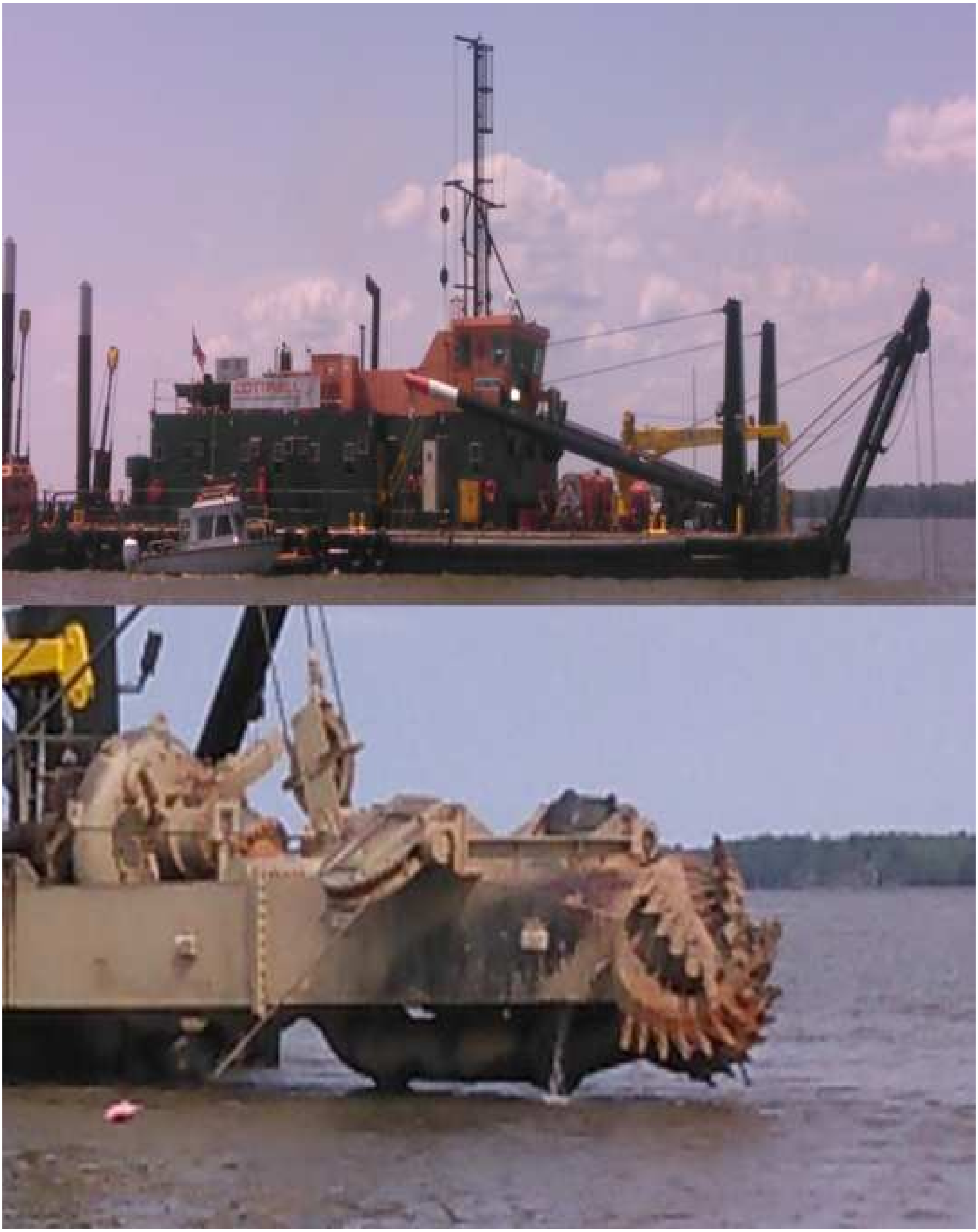

Figure 5

Click here to download Figure Fig 5.tif

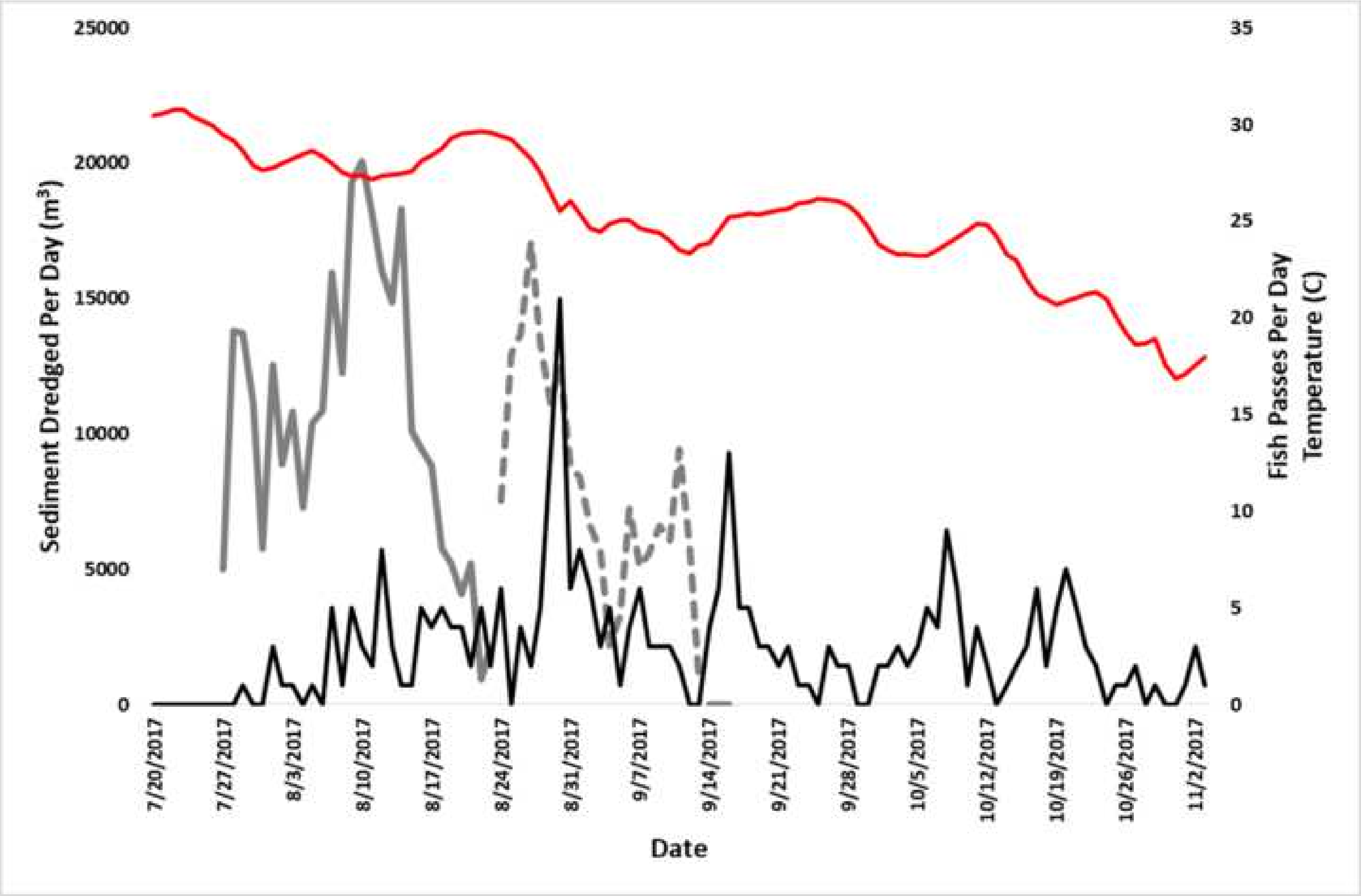

Click here to download Figure Fig 6.tif

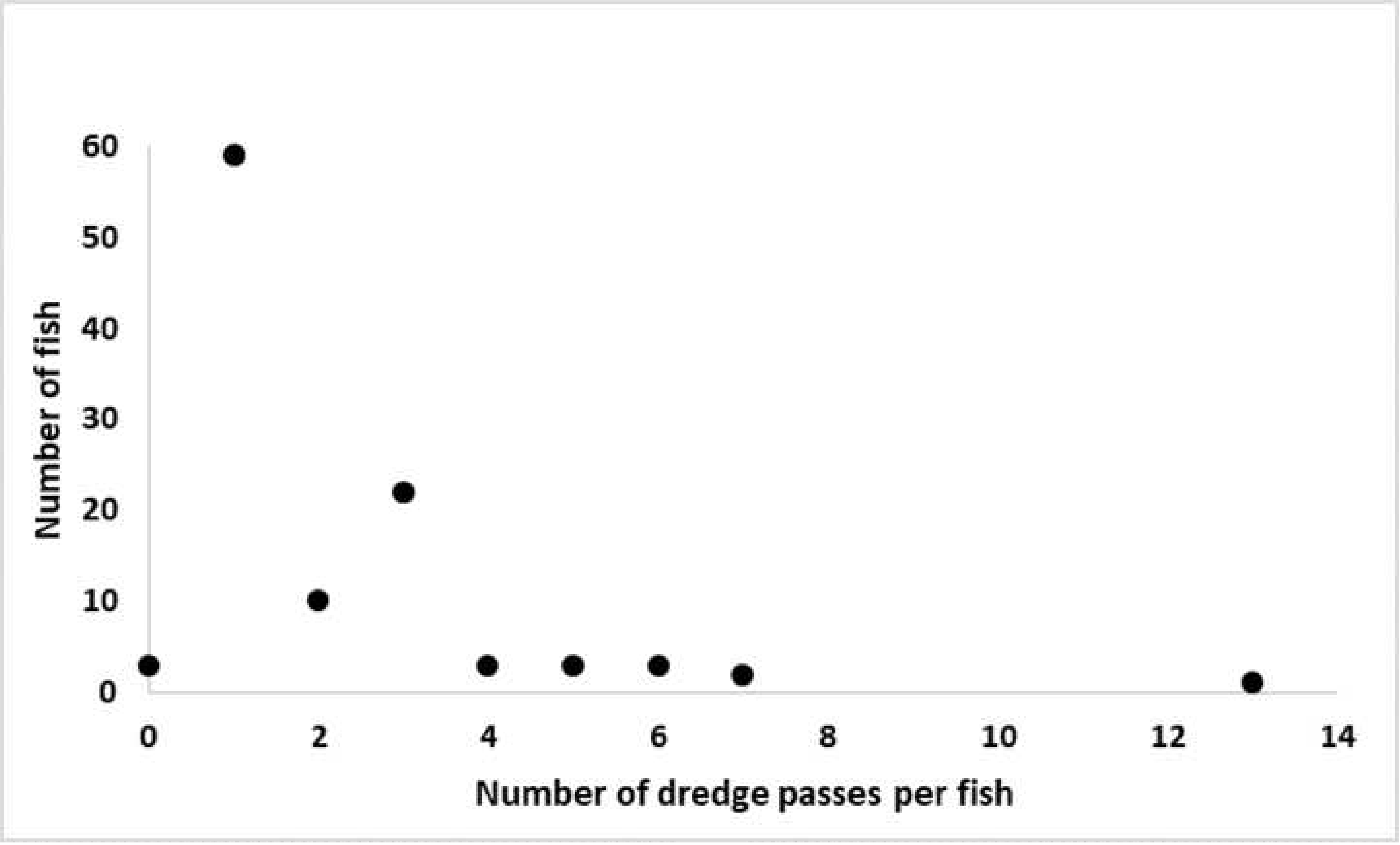

Figure 7

Click here to download Figure Fig 7.tif

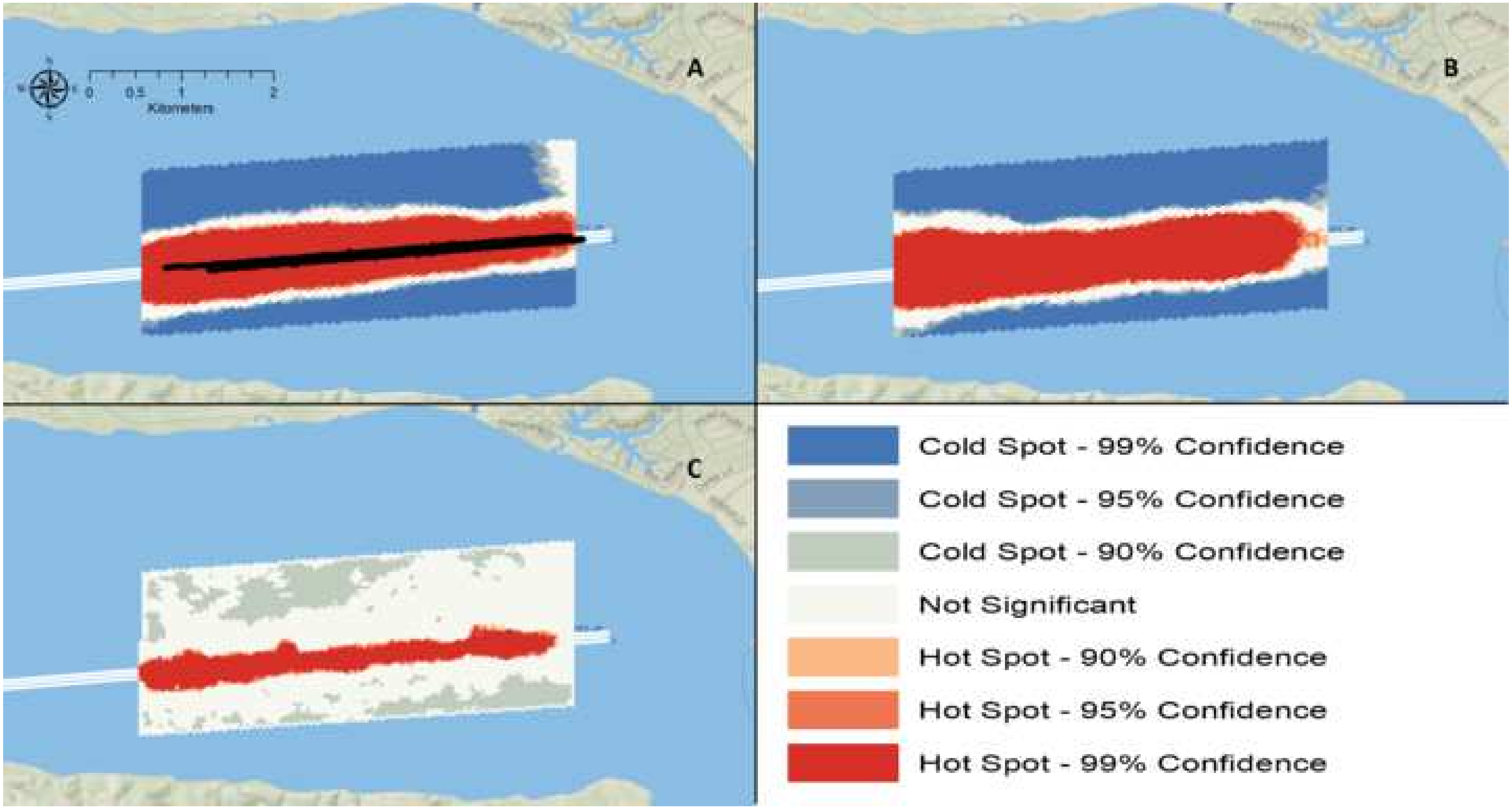

Figure 8

Click here to download Figure Fig 8.tif

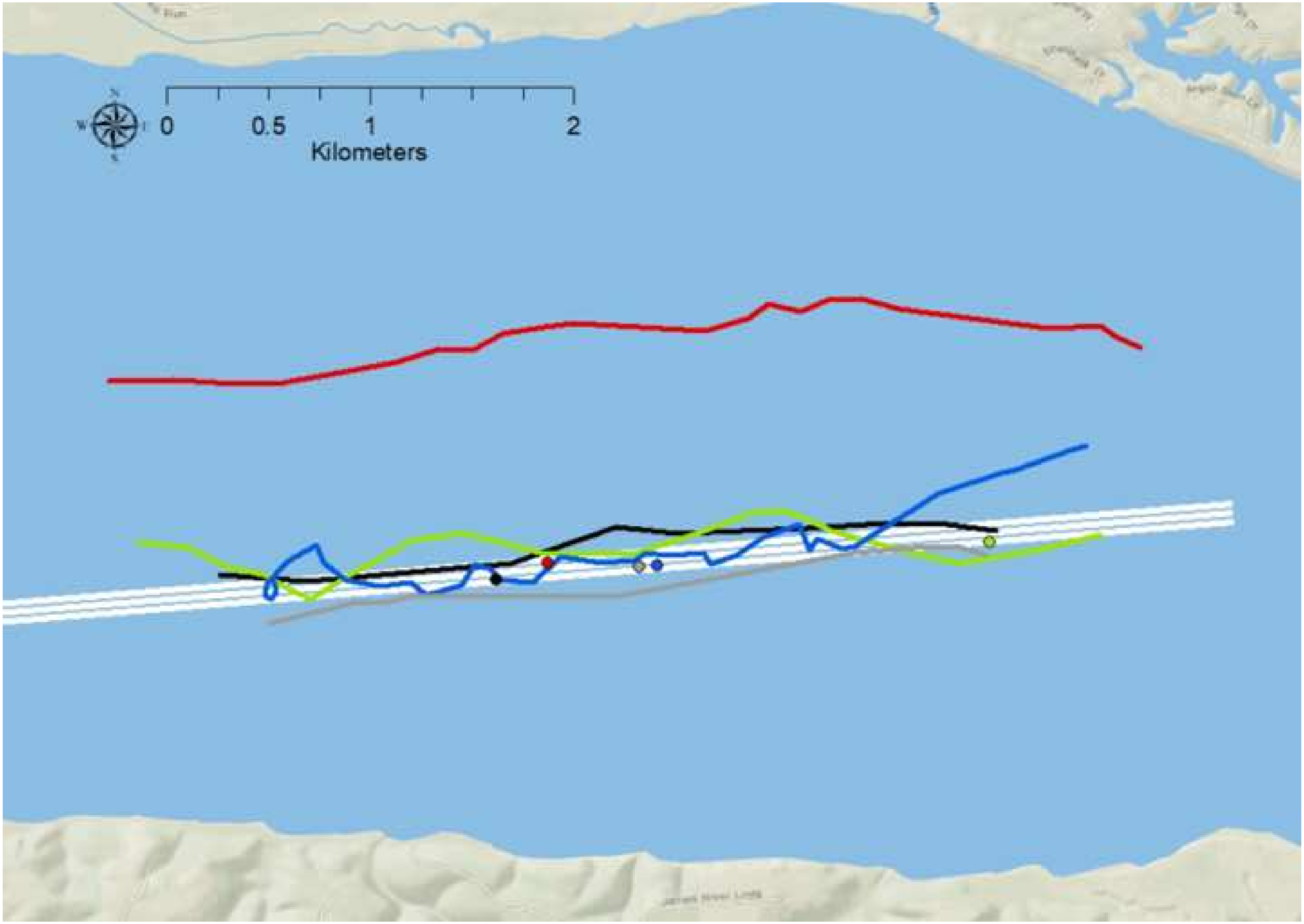

Figure 9

Click here to download Figure Fig 9.tif

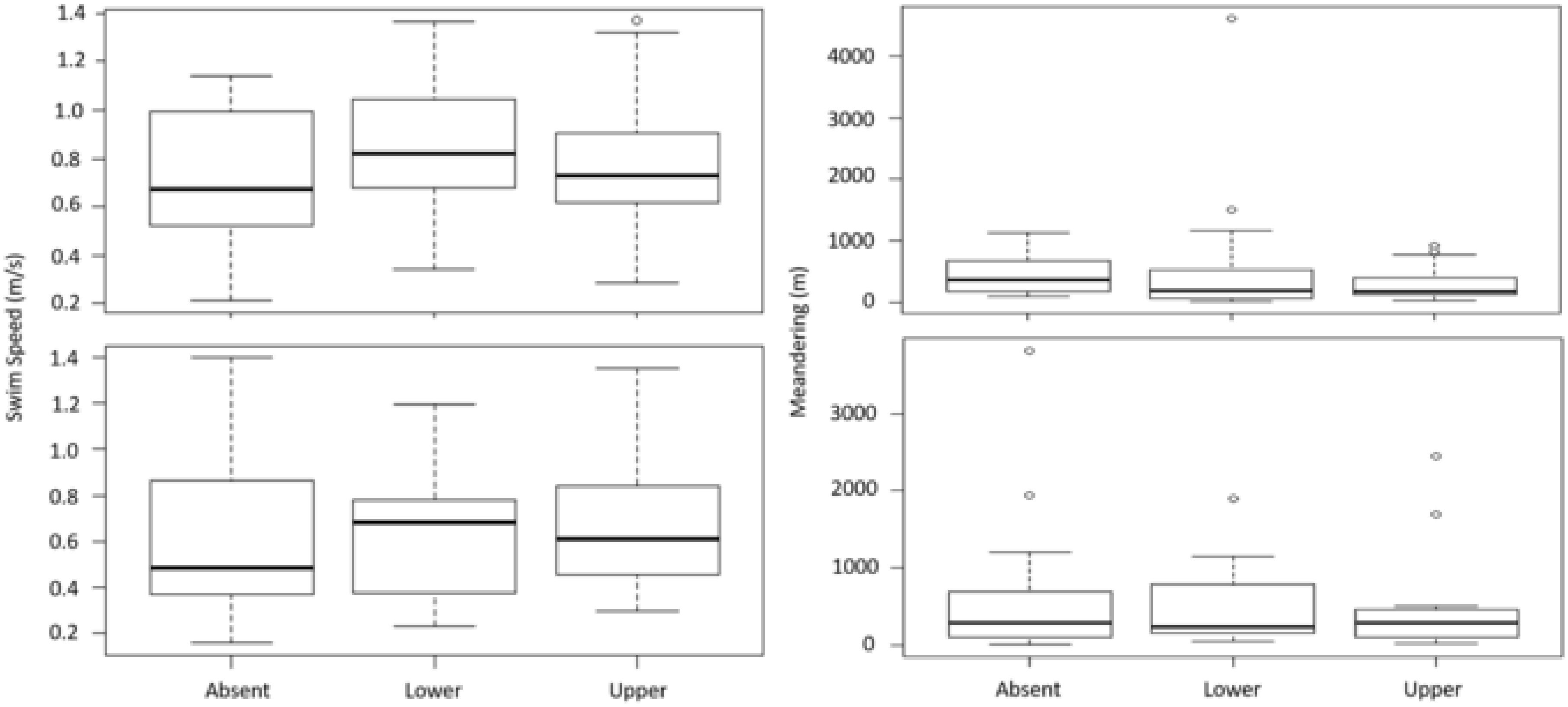

Supplement: Attachment — Submitted filename: PONE-D-19-29177_reviewer.pdf [file pone.0230029.s002.pdf]
